# Multifunctional Edible Oil-Impregnated Nanoporous Oxide Layer on AISI 304 Stainless Steel

**DOI:** 10.3390/nano13050807

**Published:** 2023-02-22

**Authors:** Kichang Bae, Minju Kang, Yeji Shin, Eunyoung Choi, Young-Mog Kim, Junghoon Lee

**Affiliations:** 1 Department of Metallurgical Engineering, Pukyong National University, Busan 48513, Republic of Korea; 2 Dongnam Division, Korea Institute of Industrial Technology, Yangsan 50623, Republic of Korea; 3 Department of Food Science and Technology, Pukyong National University, Busan 48513, Republic of Korea

**Keywords:** stainless steel, edible oil, corrosion resistance, de-icing, anti-biofouling, condensation heat transfer

## Abstract

Slippery liquid-infused porous surface (SLIPS) realized on commercial materials provides various functionalities, such as corrosion resistance, condensation heat transfer, anti-fouling, de/anti-icing, and self-cleaning. In particular, perfluorinated lubricants infused in fluorocarbon-coated porous structures have showed exceptional performances with durability; however, they caused several issues in safety, due to their difficulty in degradation and bio-accumulation. Here, we introduce a new approach to create the multifunctional lubricant-impregnated surface with edible oils and fatty acid, which are also safe to human body and degradable in nature. The edible oil-impregnated anodized nanoporous stainless steel surface shows a significantly low contact angle hysteresis and sliding angle, which is similar with general surface of fluorocarbon lubricant-infused systems. The edible oil impregnated in the hydrophobic nanoporous oxide surface also inhibits the direct contact of external aqueous solution to a solid surface structure. Due to such de-wetting property caused by a lubricating effect of edible oils, the edible oil-impregnated stainless steel surface shows enhanced corrosion resistance, anti-biofouling and condensation heat transfer with reduced ice adhesion.

## 1. Introduction

Oil-impregnated porous surfaces (or slippery lubricant-infused porous surface (SLIPS)) have been explored as a promising candidate to solve various issues related to the surface of commercial materials. The water-immiscible oil is retained in the porous structure, so that the oil inhibits a contact of external liquids to porous solid surface. Moreover, the porous structure stably immobilizes the oil against external disruption such as forced flows. Therefore, the surface shows a significant repellency to water and mobility of water droplets; thereby, various functionalities, such as water/oil separation [1,2], self-cleaning [3,4], corrosion resistance [5,6], de/anti-icing [7,8], and anti-biofouling [9,10], can be realized on commercial materials. Since the shape and dimension of pore affects not only the stability of oil in porous structure, but also the performance and durability, an appropriate technology should be employed to create a porous surface structure regarding the base material. In the case of glass, TiO_2_ and SiO_2_ nanoparticles, which are intrinsically transparent to visible light, have been used to create the porous surface structure retaining immiscible oil and maintaining its transparency [11,12,13]. Chemical conversion treatment creating nanoporous oxide structures has been used for steels and copper to enhance corrosion resistance and de-wetting performance [14,15]. Among various techniques, an anodic oxidation, which creates an oxide layer with cylindrical high-aspect-ratio dead-end pores, has been considered as the most effective method to fabricate the oil-impregnated porous surface on aluminum substrate, due to its unique pore geometry and arrangement [16]. In particular, since the shape and size of nanopores are controllable by fabrication conditions and post-treatments (e.g., pore-widening), the anodic aluminum oxide (AAO) is employed to improve the understanding on enhancement of oil stability in nanopores; and thereby, durability, which is one of the most significant issues to be solved for practical applications [17,18,19]. Moreover, the isolated pore geometry with high porosity of AAO exhibits significantly advanced durable omniphobicity and oil stability, compared to open interconnected pore structure, such as nanoneedle and pillared-surface morphology [20]. Based on such understanding, the anodic oxidation is employed to realize a omniphobic and omnicorrosion-resistant surface on stainless steel, which is used in many applications, such as chemical plants, heat exchangers, seawater desalination, marine system, and food processing [20,21,22].

Despite such efforts to extend application fields and the life-time of oil-impregnated porous surfaces, another issue for real application is the safety of lubricants (or oils) to the human body and ecosystem. In particular, even though the perfluorinated liquids with immiscibility to water and hydrocarbon liquids have been demonstrated to show superior and unique surface performances, they caused several issues in safety, due to their difficulty in degradation and bio-accumulation [23,24,25]. Therefore, the U.S. Environmental Protection Agency classifies fluorinated materials as ‘‘emerging contaminants”. Hence, research to find alternatives of perfluorinated liquids are important in the real application of lubricant-impregnated surfaces [26,27].

In this study, we fabricated the multifunctional oil-impregnated nanoporous stainless steel without using the fluorocarbon compounds, such as perfluorinated lubricants, PTFE, or FDTS coatings. Instead of perfluorinated lubricants, degradable various edible oils, which have fewer potential issues in the human body and ecosystem, are used to create a lubricant layer on the nanoporous surface. A fatty acid (i.e., stearic acid), which is also degradable in nature and a friend to human body, is employed to hydrophobize the nanoporous anodic oxide of stainless steel in order to improve chemical affinity between the edible oil and porous structure. In order to demonstrate the multifunctionalities of the edible oil-impregnated surface, de-wetting to water, corrosion resistance, de-icing, condensation heat transfer, and anti-biofouling were evaluated. Then, we discussed the potential of an edible oil-impregnated nanoporous surface as an alternative to perflurianted lubricant-impregnated surfaces by comparing the performance reported in previous papers.

## 2. Materials and Methods

### 2.1. Pretreatment of Stainless Steel

A mirror-finished AISI 304 stainless steel (SS304) sheet (thickness: 1 mm) cut into 30 × 30 mm^2^ size was used as a substrate for surface treatment. In order to remove contamination and native oxide film, the specimen was degreased and activated in detergent aqueous solution for 5 min and 15 wt.% HCl (Junsei Chemical, Tokyo, Japan) for 10 min, respectively. A cleaned SS304 sample was immersed in 1.65 M FeCl_3_ (Junsei Chemical, Japan) + 12.08 M HCl + 9.76 M H_2_O_2_ (Samchun Chemicals, Seoul, Republic of Korea) aqueous solution for 20 min as a chemical etching to make the surface rougher. After each treatment, the specimen was washed in deionized water with ultrasonication for 2 min; then, dried with compressed air.

### 2.2. Anodization of SS304

An anodic constant voltage of 60 V was applied to the SS304 sample in an ethylene glycol-based electrolyte with 0.1 M H_2_O + 0.2 M NH_4_F (Samchun Chemicals, Republic of Korea) at 15 °C for 5 min. The platinum mesh was used for the cathode. Then, the surface was immediately cleaned with acetone, and annealed on a hot plate at 350 °C for 1 h to stabilize the nanoporous oxide layer [21].

### 2.3. Hydrophobization and Oil Impregnation

The anodized specimen was immersed in an ethanol solution with 0.05 M stearic acid (Samchun Chemicals, Republic of Korea) for 3 h to hydrophobize the nanoporous oxide surface. Then, the sample was washed with ethanol and dried with compressed air. As a model case, we used oleic acid, which is the main ingredient of edible oil. Considering practical applications, we also used 5 different edible oils, such as olive oil, canola oil, sunflower oil, corn oil, and grape seed oil, which can be easily found in our daily life. In order to completely impregnate the edible oil into nanopores of anodic oxide, we used the solvent-exchange method. First, the hydrophobized sample with nanoporous anodic oxide is immersed in acetone for 20 min with ultrasonication. Then, the sample is transferred to edible oil without drying the wetted acetone on the surface and immersed in edible oil for 30 min with ultrasonication. We fabricated various type of samples to examine performances; thus, the name of samples regarding the treatments and oil types were summarized in Table 1.

### 2.4. Material Characterizations

The surface and cross-section of the nanoporous anodized layer were observed using a field-emission scanning electron microscope (FE-SEM, MIRA 3, TESCAN, Brno, Czech Republic). Chemical analysis for hydrophobic coating with stearic acid was conducted using Fourier-transform infrared (FT-IR, VERTEX 70, Bruker, Billerica, MA, USA) analysis in the wavenumber range of 500 to 4000 cm^−1^.

To evaluate the wettability and mobility of liquid droplets on the specimen, the static contact angle and the contact angle hysteresis of a sessile water droplet (5 μL) were measured using a goniometer system (SmartDrop, Femtobiomed, Seongnam, Republic of Korea). To observe the sliding of water droplet on the oil-impregnated surfaces, a sessile water droplet was gently deposited on the pre-inclined (3°) surface.

The corrosion resistance of oil-impregnated anodized stainless steel was evaluated by a potentiodynamic polarization test in a 1.0 M HCl solution at room temperature with a three-electrodes flat cell and potentiostat (VersaSTAT3, AMETEK, Berwyn, PA, USA). The saturated calomel electrode (SCE) and platinum mesh were used as reference and counter electrodes, respectively. Before the potential scan, the specimen was immersed in a 1.0 M HCl solution for 30 min to stabilize the open-circuit potential (OCP). The potential was scanned from −650 mV to 800 mV vs. OCP at 2 mV/s rates. To minimize the experimental error in electrochemical corrosion analysis (i.e., potentidynamic polarization test), we used seven samples fabricated by each condition. Excluding potentiodynamic polarization curves with the maximum and minimum corrosion current density, five corrosion current densities were averaged. Then, potentiodynamic polarization curves, which have the most similar values (i.e., corrosion current density), on average, were shown as representative results.

The ice-adhesion force was measured using a horizontal push method in the customized experimental setup at the surface temperature of −5 °C. A polytetrafluoroethylene (PTFE) cube was installed on the specimen. When the surface temperature of the specimen reached to −5 °C, the PTFE cube was filled with 1 mL of distilled water and cooled for 30 min to be completely frozen. Then, the maximum shear load was measured by pushing the PTFE cube with a push–pull gauge. The de-icing force, which is the maximum shear load to move the PTFE cube with adhere ice, was measured a total of 5 times and then averaged.

For the anti-biofouling test, we used *Pseudomonas aeruginosa* bacteria (KCTC 1637), which can be best seen in daily life and form biofilm. The sample was immersed in 4 μL of Tryptic soy broth (TSB; Difco Laboratory Inc., Detroit, MI, USA) solution with bacteria; then, the bacteria were cultured in an incubator at 35 °C for 24 h. After that, each sample was transferred to another clean Petri dish and rinsed with distilled water 3 times to remove the contaminated TSB solution. The cleaned sample was stained with 0.1% crystal violet dye for 15 min at room temperature; then, the stained sample was rinsed with distilled water 3 times to remove residual dye on the sample surface. The dye-bonded bacteria on the sample surface were solubilized using 95% ethanol; then, the quantitative nature of stained cells was measured by optical density (OD) at the wavelength of 570 nm using a multi-mode microplate reader. Each experiment was carried out five times [28].

The condensation heat transfer was evaluated by the customized-manufactured experimental setup. The specimen was fixed to the copper (Cu) meter bar attached to cooling block, which is cooled by water circulation at 20 °C. Four T-type thermocouples are inserted in the Cu meter bar with 1 cm spacing, so that the heat transferred from the sample surface to the cooling block can be estimated by the temperature gradient in the meter bar and the thermal conductivity of the meter bar. The sample surface is exposed to 99 °C, humid conditions. The temperatures in the meter bar are stabilized by operating more than two hours. A more detailed setup for the condensation heat-transfer test can be found in the Supplementary Information (Figure S1).

## 3. Results and Discussion

**Figure 1:**
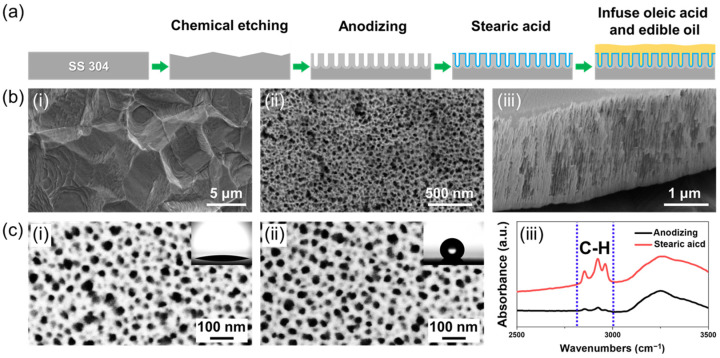
(**a**) Schematic procedure for sample preparation with chemical etching, anodic oxidation, hydrophobizing, and oil impregnation. (**b**) SEM image of (**i**) chemically etched SS304 surface, (**ii**) surface, and (**iii**) cross-section of the anodic oxide of SS304. (**c**) SEM image of (**i**) before and (**ii**) after the hydrophobizing by immersing in stearic acid, and (**iii**) FT-IR spectra of anodic oxide surface with and without hydrophobizing (i.e., immersing in stearic acid).

In order to create the nanoporous oxide layer, which is necessary for oil-impregnation and the retention of oil on the surface, a chemical etching, anodic oxidation, and hydrophobic coating are employed. Figure 1a shows the schematic fabrication process of the edible oil-impregnated nanoporous oxide surface on SS. The native oxide layer on SS is removed and the surface is etched to create a micro-scale rough surface morphology (Figure 1(b-i)) by chemical etching. After that, the sample is anodized in ethylene glycol-based solution. As a result of chemical etching and anodic oxidation, a porous oxide layer (thickness: 2 μm) with cylindrical pores (diameter: 25 ± 8 nm) is formed on SS304 (Figure 1(b-ii,b-iii)).

The nanoporous oxide structure, which is naturally hydrophilic, significantly increases surface roughness compared to the bare surface; thus, the anodized SS304 shows an extremely low static contact angle of 11.3 ± 2° (insert in Figure 1(c-i)). Moreover, the wettable nanoporous oxide surface to water precludes the observation of water/solid contact line and measurement of dynamic contact angle (i.e., advancing and receding contact angle). Due to this high wettability of the nanoporous oxide surface, the hydrophobizing surface coating is necessary for realization of the slippery oil-impregnated surface [16]. The hydrophilic oxide surface is hydrophobized in stearic acid solution that does not contain fluorine, causing serious environmental problems and health issues. Figure 1(c-i,c-ii) show the SEM images of the nanoporous anodic oxide of SS before and after hydrophobizing in stearic acid, respectively. The coating in stearic acid shows a negligible effect to the nanoporous oxide structure; however, the static contact angles significantly increase from 11.3 ± 2° to 141.1 ± 1°, indicating that the wettability of surface to the water decreases to become hydrophobic. In addition, due to the hydrophobizing in stearic acid, an enhancement of peaks for C-H vibration in FT-IR (Figure 1(c-iii)) can be observed in the wavenumber range of 2800–3100 cm^−1^. These results indicate that the hydrophobic hydrocarbon molecular is bonded to the oxide surface to form a monomolecular layer in the stearic acid solution, so that the chemical affinity between nanoporous oxide surface and edible oil, and the repellency to water can be enhanced.

**Figure 2:**
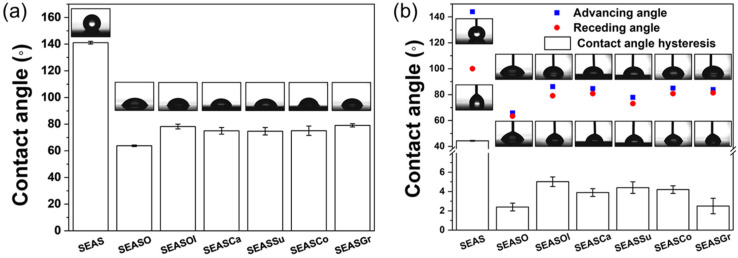
(**a**) Static contact angle and (**b**) advancing/receding contact angle, and contact angle hysteresis of water droplet.

The edible oil was impregnated into the hydrophobized nanoporous anodic oxide of SS; then, the wettability and mobility of water droplet on surfaces were examined (Figure 2). In addition, sequential images of water droplets on pre-inclined (3°) surfaces were shown in Figure S2. Due to the hydrophobizing in stearic acid solution, the nanoporous anodic oxide surface (SEAS) shows a hydrophobicity with a static contact angle of 141°. In addition, the nanoporous oxide structure also contributes to a high static contact angle, following the Cassie-Baxter rendering and reduction of contact area of the water droplet to a solid surface [29]. However, the disconnected pore arrangement with the continuous oxide surface of anodic oxide strengthens the pinning of the liquid/solid contact line [30,31]. Therefore, even though the SEAS shows a static contact angle of 141.1° and contact angle hysteresis of 44.3°, the water droplet is immobile on pre-inclined surface at 3° (SEAS, Figure S2). The impregnation of oleic acid and edible oils into nanoporous anodic oxide decreases the static contact angle; however, the mobility of the water droplet is significantly increased with a significant decrease of contact angle hysteresis (less than 6.0°) and sliding angle (less than 3°, Figure S2). Such a low contact angle hysteresis and sliding angle of the water droplet is not realized on the edible oil-impregnated hydrophobic SS304 surface without the nanoporous oxide structure. This is because the oleic acid on hydrophobized SS304 and SS304 with chemical etching forms an oil droplet, while the oleic acid is extremely wettable on a hydrophobized nanoporous surface to form a lubricating layer (Figure S3). These results also imply that the hydrophobic nanoporous oxide layer with cylindrical pores not only improves the wettability of edible oil to solid surface for repellency to water, but also enhances the retentivity of oil [21].

The types of edible oil do not show any significant differences in contact angle hysteresis and sliding angle, while the static apparent contact angle on the oleic-acid-impregnated surface (~78.2°) is lower than those of the edible oil-impregnated surfaces (e.g., olive, canola, sunflower, corn, and grape seed oil). The edible oil is generally a mixture of oleic acid (surface tension: ~32 mN/m) and linoleic acid (surface tension: ~25 mN/m); thus, the surface tension of edible oil is lower than that of oleic acid. The shape of the water droplet on oil-impregnated surface is affected by the interfacial force balance among the oil, liquid, and air; instead of the solid, liquid, and air for the cases without oil impregnation [32,33,34]. Therefore, the water droplet on edible oil-impregnated surfaces shows a more spherical shape than the case of oleic acid. Moreover, Krytox lubricants (16–20 mN/m) generally used for SLIPS have a lower surface tension than edible oils; thus, the Krytox-lubricants-impregnated surface shows a higher apparent contact angle (~100°) than the surface fabricated in this study [20,35]. Despite the difference in the apparent contact angle of the water droplet by the surface tension of lubricants below the droplet, the sliding angle (less than 3°) and contact angle hysteresis (less than 7.0°) of the water droplet on the edible oil-impregnated surfaces is almost similar. These results indicates that the oil is lubricating between the water droplet and porous oxide; thus, the multifunctionalities of SLIPS (using Krytox (or perfluorinated) oils) can be also realized using the edible oils.

**Figure 3:**
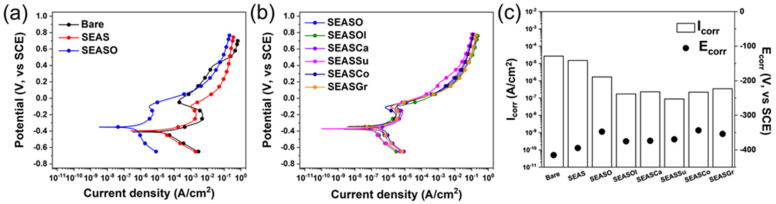
Potentiodynamic polarization curves in 1.0 M HCl of (**a**) bare, SEAS, and SEASO; (**b**) SEASO, SEASOl, SEASCa, SEASSu, SEASCo, and SEASGr. (**c**) Estimated corrosion current density (I_corr_) and potential (E_corr_).

The fluorocarbon lubricant impregnated in the nanoporous surface oxide layer inhibits the direct contact of corrosive liquid to the base metal, so that the surface significantly enhances the corrosion resistance by ~99.99% of the protection efficiency [20]. In order to verify the efficiency of the edible oil-impregnated surface for this anti-corrosion, a potentiodynamic polarization test was conducted in a 1.0 M HCl solution. The obtained potentiodynamic polarization curves and estimated corrosion potential/current density were shown in Figure 3. In this study, we used a HCl solution, which has lower pH than NaCl solution; thus, the current response in anodic polarization is much more reliable and reproducible. However, the 304 stainless steel was not shown in the passivation region in anodic branch, unlike the case of the NaCl solution. Due to the hydrophobizing and anodic oxidation of stainless steel (SEAS) enhancing the de-wetting of water, the corrosion potential increased to −0.39 V from −0.41 V (bare SS304) and the corrosion current density decreased to 1.50 × 10^−5^ A/cm^2^ from 2.65 × 10^−5^ A/cm^2^ (bare SS304). Such reduction in corrosion current density is contributed by the formation of the Cassie-Baxter state between the corrosive liquid and porous surface, which impregnates air within the pores of the oxide layer under aqueous solution. The impregnation of edible oil (i.e., oleic acid) in this hydrophobic nanoporous oxide layer further decreases the corrosion current density and increases corrosion potential, indicating the better corrosion resistance than the case without oil impregnation. These results indicate that the oleic acid impregnated in the nanopores are more effective to inhibit the penetration of corrosive liquid toward base stainless steel than the air, which is entrapped in nanopores rendering the Cassie-Baxter state [29]. The nanoporous oxide layers with conventional edible oils (e.g., olive, canola, sunflower, grape seed oils) show a slightly lower corrosion current density and similar potential with the surface compared to the case of oleic acid, indicating that the impregnation of conventional edible oils is also effective for anti-corrosion in a manner the same as oleic acid. Moreover, the types of edible oils did not show significant differences in corrosion current density and corrosion potential. However, the protection efficiency of the edible oil-impregnated surface (~99.2%) is lower than the case with fluorocarbon lubricants (~99.9%) [21]. This difference in protection efficiency is caused by the surface tension of impregnated lubricants. The surface tension of oleic acid is higher than edible oils; thus, the static contact angle on oleic acid-impregnated surface is higher than the cases with edible oil, indicating that the water can more easily contact the nanoporous surface structure. Since the corrosion resistance of oil-impregnated surface depends on the contact of corrosive media to the surface porous structure, the oleic acid-impregnated surface shows a higher corrosion current density than the cases of edible oils. For the same reason, the edible oil-impregnated surfaces show a slightly lower protection efficiency than the nanoporous surface impregnated with fluorocarbon lubricants, which have lower surface tensions than edible oils. Nevertheless, it should be noted that the impregnation of edible oil for anodized SS304 reduces the corrosion current density by more than two orders of magnitude compared to bare SS304, indicating a significant improvement in corrosion resistance.

**Figure 4:**
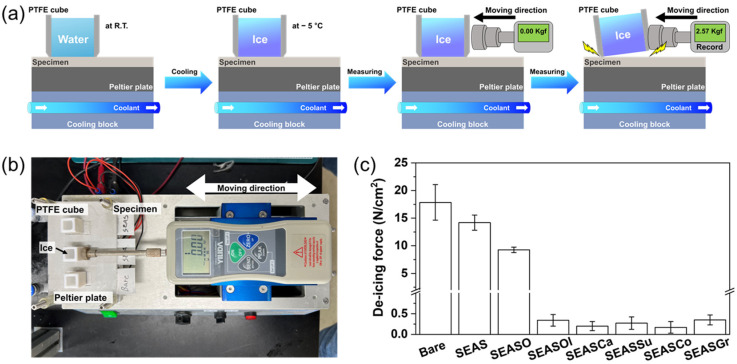
(**a**) Schematic images of measuring process for de-icing force. (**b**) Measurement setup for de-icing force, and (**c**) de-icing force on stainless steel surfaces with various treatments.

Icephobicity is one of the representative properties of lubricant-impregnated surfaces. In previous reports, the fluorocarbon lubricant-impregnated nanoporous surfaces show a significantly low adhesion force (~ 0.1 N/cm^2^) between the ice cube in the shear-detaching test [36]. Therefore, in order to find out whether the edible oil-impregnated surfaces also have such icephobicity, we also evaluated the ice-adhesion force. Details of the procedure and test setup for the shear-detaching test of ice were shown in Figure 4a,b, respectively. The maximum load of the push–pull gauge while pushing the ice cube attached to the surface was recorded as the adhesion force of ice. The adhesion force on the bare SS304 was 17.85 N/cm^2^. Due to the realization of the hydrophobic surface (SEAS), rendering the Cassie-Baxter state, the adhesion force of ice decreases to 14.19 N/cm^2^. However, the decrease of ice adhesion force by anodic oxidation and following hydrophobization is not so significant compared to the change of static contact angle, because the ice forms an interlocking with the top of the nanoporous surface [37]. The impregnation of oleic acid to a nanoporous hydrophobic surface (SEASO) further decreases the ice adhesion force to 9.27 N/cm^2^. However, the edible oil-impregnated surfaces show a significantly low ice-adhesion force less than ~1.0 N/cm^2^, which indicate the ice cube is easily moved by a slight push. Even though the oleic acid sufficiently inhibits the direct contact of water to a nanoporous surface, it can freeze at below 15°; thus, the dramatic decrease of ice adhesion was not achieved with oleic acid. Meanwhile, the edible oils did not freeze at the temperature of the testing surface (~−5 °C). Therefore, the edible oils inhibit the formation of interlocking between ice and the nanoporous surface, so that the ice can be easily moved by a weak shear force [38,39,40]. Fluorocarbon lubricant-impregnated surfaces have also been reported to show a significant low adhesion of ice (~0.1 N/cm^2^) [36]. Thus, as long as the lubricants do not freeze, the edible oil-impregnated surfaces would have similar deicing performance to the surfaces with fluorocarbon lubricants.

**Figure 5:**
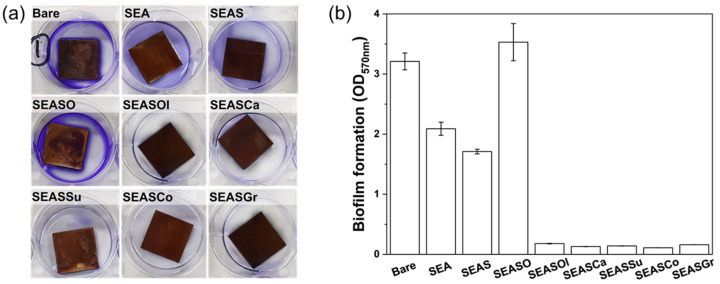
(**a**) Images of dyed solution with sample and (**b**) optical density of dyed *Pseudomonas aeruginosa*.

Since the omniphobic lubricant-infused porous surface is introduced, its anti-bioufouling and inhibition of biofilm formation have been widely explored [22,41,42]. The bacterial medium is immiscible to the infused lubricant; thus, the bacterial attachment and formation of biofilm are significantly inhibited. We also tested such anti-bacterial performance of the edible oil-impregnated surface by incubating *Pseudomonas aeruginosa* on surfaces for 24 h. Crystal violet was used to stain the attached biofilm cells on surfaces; then, the optical density (OD) of the stained bacterial solution was measured at 570 nm. Figure 5 shows the images of stained bacterial solution with the sample and measured OD at 570 nm for crystal violet absorbance, which is proportional to the attached biomass. The OD of bare SS304 is 3.21, and it decreased to 2.09 by anodic oxidation. Further decrease of OD to 1.71 was shown for hydrophobized anodic oxide. Such reduction in biofilm is attributed to a decrease in surface area by anodic oxidation and adhesion force by hydrophobizing with the low-surface-energy material [43]. Contrary to our expectations, the oleic acid-impregnated surface (SEASO) showed ~10% higher OD (3.53) than bare SS304. The membrane forming the cell of *Pseudomonas aeruginosa* has a similar molecular structure with oleic acid, which is monosaturated fatty acid; thus, the *Pseudomonas aeruginosa* adheres better upon contact with oleic acid. Thus, the *Pseudomonas aeruginosa* was more cultivated on the oleic acid-impregnated surface. However, the edible oils also include linoleic acid, which is known to inhibit biofilm formation under static and continuous conditions without inhibiting the growth of *Pseudomonas aeruginosa* [44]. Therefore, the edible oil-impregnated surface shows significantly reduced OD less than 0.2, which indicates the inhibition of biofilm formation by more than 93.8% compared to bare SS304. Comparing to SEAS, which is a hydrophobic nanoporous surface, more than 88.3% of biofilm formation is reduced by the edible oil-impregnation. Such reduction in biofilm formation by the edible oil-impregnation is less significant than the perfluorinated lubricant (i.e., Krytox oils), which showed more than 95% reduction for *Pseudomonas aeruginosa*, *Staphylococcus aureus*, and *Escherichia coli* [45]. These difference in anti-biofouling performance is attributed to the composition of edible oil containing oleic acid, which allows the adhesion of bacterial to oily surfaces. Nevertheless, it should be noted that the edible oil also contains linoleic acid, which inhibits the biofilm formation, and the oily surface is immiscible to an aqueous medium; thus, the edible oil-impregnated nanoporous surface has an anti-biofouling performance.

**Figure 6:**
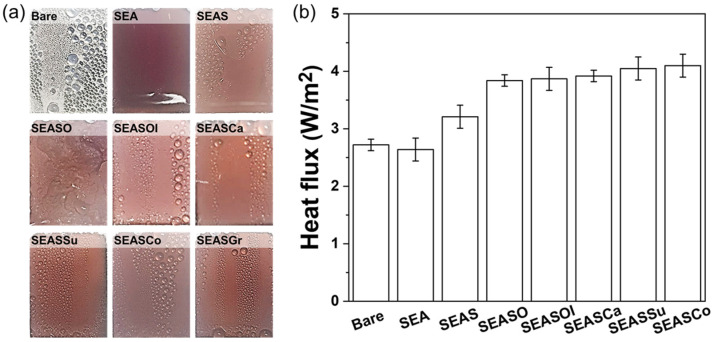
(**a**) The appearance of condensed water on each specimen and (**b**) estimated heat flux by condensation on sample surface.

When the highly de-wettable surface is cooled, the water droplets condense, and the water droplets are easily removed from the highly water-mobile surface by gravity. Therefore, the cold solid surface is continuously exposed to hot and humid conditions; then, a condensation heat transfer, which is important in water harvesting and desalination, environmental control, and power generation systems, is significantly enhanced. In particular, the condensed water droplet has a larger contact area on the lubricant-impregnated surfaces than nanoporous superhydrophobic/hydrophobic surfaces; thus, the transfer of latent heat during the condensation is higher for the lubricant-impregnated surfaces. Therefore, the lubricant-impregnated surfaces are promising candidates for improving the condensation heat transfer in various engineering systems. We also evaluated the condensation heat transfer of edible oil-impregnated nanoporous oxide of stainless steel using a self-fabricated test setup (see Figure S1). The temperatures of humid ambient and coolant are maintained at 20 and 99 °C, respectively. Then, the measured temperature gradient in the meter bar is shown in Figure S4 and Table S1. The heat flux by condensation heat transfer from the sample surface is calculated with the temperature gradient and thermal conductivity of the meter bar (pure copper 99.9%, 391.1 W/m∙K). A detailed method to determine the heat flux can be found in the Supporting Information. The heat flux on bare SS304 is 2.7 W/m^2^; then, it decreases to 2.6 W/m^2^ by the anodic oxidation. This is because the bare shows dropwise condensation (Figure 6a), which is favorable to expose the cold solid surface, while the water condensation on the nanoporous anodic oxide surface shows a filmwise mode. The hydrophobizing with stearic acid on nanoporous anodic oxides changes the condensation mode to dropwise; the heat flux increased to 3.2 W/m^2^. However, even though the surface is hydrophobized (SEAS), the water droplet still has a significant contact angle hysteresis, indicating the immobility of the droplet (Figure 2); thus, the heat flux is not significantly increased. The edible oil impregnation into hydrophobic nanoporous oxides enhanced the mobility of water droplets (Figure 6); thus, the oleic acid and edible oil-impregnated surfaces show significantly increased heat flux to ~4.0 W/m^2^, which is 25% and 48% higher than the hydrophobized surface (SEAS) and bare SS304, respectively. The enhancement of heat flux on the perfluorinated lubricant-impregnated surface was reported to be 50% compared to the Cu substrate at a sample temperature of 23 °C and an ambient temperature of 66 °C [46]. In our case, the temperatures of the sample and ambient are 23 and 99 °C, respectively. Thus, it is not fair to compare directly with our results. If it is considered that the heat flux by water condensation increases with the increase of temperature differences between the sample surface and ambient; the condensation heat transfer on the perfluorinated lubricant-impregnated surface would be higher than edible oil-impregnated surfaces. However, the liquid lubricant in the nanoporous structure is not perfectly immobile; thus, it can be depleted in the porous structure by moving the water droplet on the surface and releasing to an ambient environment with condensed water. In this case, the edible oil can mitigate the impact to environmental pollution, which can be an advantage compared to perfluorinated lubricants.

In this study, we fabricated the multifunctional de-wetting surface on stainless steel without using fluorocarbon materials and liquids, with which we have serious issues in human body and nature. The nanoporous stainless steel surface with edible oil showed similar functionalities to the surfaces with fluorocarbon compounds. However, such superior multifunctionalities of the liquid lubricant-impregnated surface can be degraded by a depletion of lubricant in the porous structure [47,48,49]. Moreover, a repeating de-icing test, which also causes a loss of liquid lubricant on the surface, gradually increases the ice adhesion strength. Moreover, some interconnected porous structures easily allow the loss of lubricants against shear flow, which causes the redistribution of the liquid lubricant layer [50], so that the superior de-wetting performance can be eliminated. Even though such limitations of a liquid lubricant-impregnated porous surface are reported for perfluorinated lubricants, the edible oil-impregnated surface cannot avoid these issues and limitations in durability. Therefore, despite the advantage in practical application of edible oil-impregnated surfaces with eco and human body friendship, the limitation in the depletion of liquid lubricant in a porous surface structure should be overcome.

## 4. Conclusions

Edible oil-impregnation into the hydrophobized nanoporous anodic oxide of SS304 significantly increases the mobility of water droplets on the surface. Enhanced de-wetting of the nanoporous surface by edible oil-impregnation also realized multifunctionalities similar to the perfluorinated lubricant-impregnated surfaces, which are generally used. The edible oil-impregnated nanoporous anodic oxide surface shows a significantly improved corrosion protection up to 99.2% and reduced ice adhesion by 5.6% compared to bare surface. Due to the de-wetting to aqueous media and linoleic acid, the edible oil-impregnated surface inhibits the formation of biofilm by *Pseudomonas aeruginosa*. Furthermore, a dropwise condensation and high mobility of the condensed water droplet is caused on the edible oil-impregnated surface, thereby improving condensation heat transfer performance by more than 48%. It has been confirmed that the slippery de-wettable liquid-impregnated porous surfaces are well realized with multifunctionalities; even the eco-friendly edible oils are impregnated in a nanoporous structure instead of the perfluorinated lubricant.

## Figures and Tables

**Table 1:** Sample name regarding the treatments and oil types.

Sample Name	Bare	SEA	SEAS	SEASO	SEASOl	SEASCa	SEASSu	SEASCo	SEASGr
Chemical etching	-	O	O	O	O	O	O	O	O
Anodic oxidation	-	O	O	O	O	O	O	O	O
Hydrophobizing	-	-	O	O	O	O	O	O	O
Oil impregnation	-	-	-	Oleic acid	Olive oil	Canola oil	Sunflower oil	Corn oil	Grape seed oil

## Data Availability

Not applicable.

## Supplementary Materials

The following supporting information can be downloaded at: https://www.mdpi.com/article/10.3390/nano13050807/s1, Figure S1: Schematic images of (a) the test setup and (b) cross-section of copper meter bar with sample attachment; Figure S2. Sequential images of sliding water droplet on oil-impregnated nanoporous oxide surfaces (pre-inclined 3°) on bare stainless steel (Bare); stainless steel with chemical etching and anodic oxidation (SEA); stainless steel with chemical etching, anodic oxidation, and hydrophobizing (SEAS); oleic acid (SEASO); olive oil (SEASOl); canola oil (SEASCa); sunflower oil (SEASSu); corn oil (SEASCo); and grape seed oil (SEASGr)-impregnated stainless steel surface with chemical etching, anodic oxidation, and hydrophobizing; Figure S3. Contact angle of the oleic acid on stainless steel with chemical etching (SE); stainless steel with chemical etching and anodic oxidation (SEA); and stainless steel with chemical etching, anodic oxidation, and hydrophobizing (SEAS); Figure S4. Temperature profile in the meter bar during the condensation test; Table S1. Estimated temperature gradient from measured temperature in meter bar during condensation heat transfer.
