# Supplementary material for: Multifunctional Edible Oil-Impregnated Nanoporous Oxide Layer on AISI 304 Stainless Steel
Source: Nanomaterials (Basel). 2023 Feb 22;13(5):807. doi: 10.3390/nano13050807 (PMC10005306; doi:10.3390/nano13050807)
Supplement: Supplementary file 1 [file nanomaterials-13-00807-s001.zip › nanomaterials-2213636-supplementary.pdf]

Multifunctional Edible Oil-Impregnated Nanoporous Oxide Layer on AISI 304 Stainless Steel

Kichang Bae [1,†], Minju Kang [1,†], Yeji Shin [1], Eunyoung Choi [2], Young-Mog Kim [3] and Junghoon Lee [1,*]

[1] Department of Metallurgical Engineering, Pukyong National University, Busan 48513, Republic of Korea;
kichbae@pknu.ac.kr (K.B.); minju2930@pukyong.ac.kr (M.K.);
yeahhji_s98@pukyong.ac.kr (Y.S.)
[2] Dongnam Division, Korea Institute of Industrial Technology, Yangsan 50623, Republic of Korea;
cossud@kitech.re.kr
[3] Department of Food Science and Technology, Pukyong National University, Busan 48513, Republic of Korea; ymkim@pknu.ac.kr
* Correspondence: jlee1@pknu.ac.kr
† These authors contributed equally to this work.

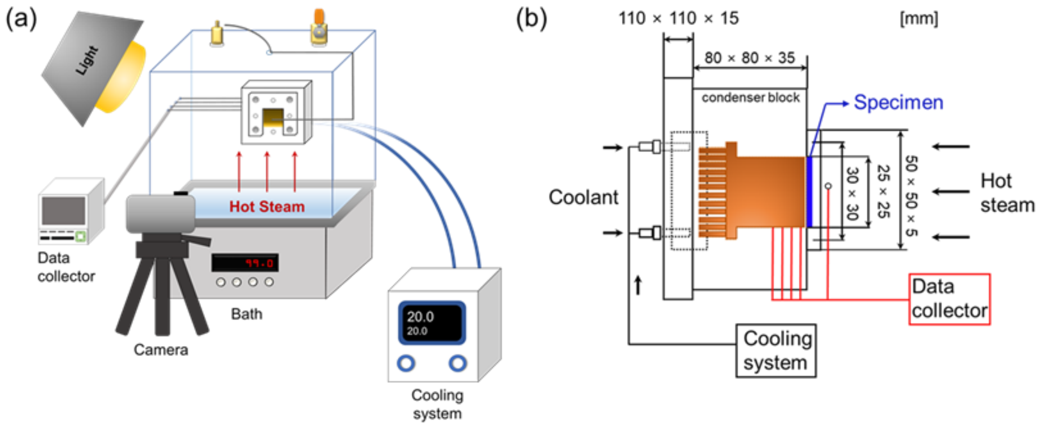

Figure S1. Schematic images of (**a**) the test setup and (**b**) cross-section of copper meter bar with sample attachment.

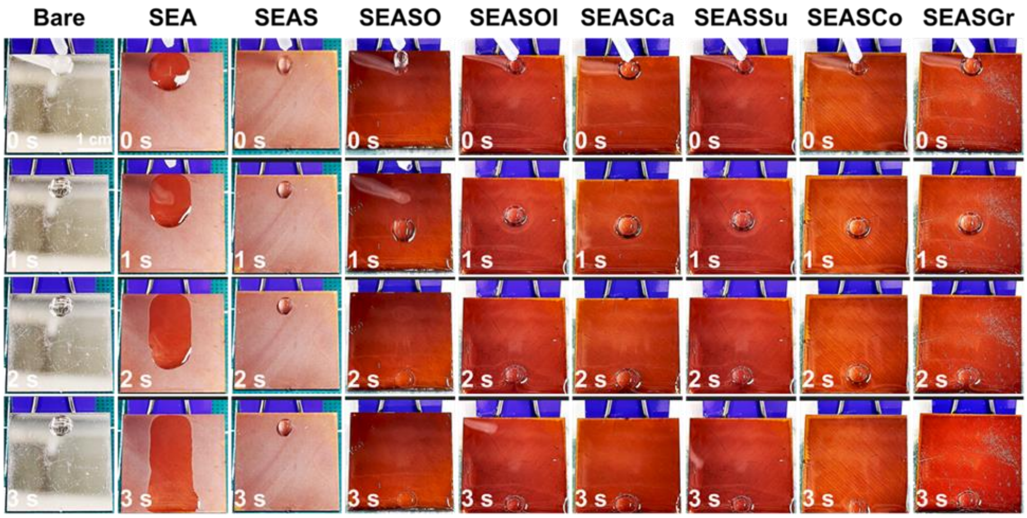

Figure S2. Sequential images of sliding water droplet on oil-impregnated nanoporous oxide surfaces (pre-inclined 3°) on bare stainless steel (Bare), stainless steel with chemical etching and anodic oxidation (SEA), stainless steel with chemical etching, anodic oxidation and hydrophobizing (SEAS), oleic acid (SEASO), olive oil (SEASOl), canola oil (SEASCa), sunflower oil (SEASSu), corn oil (SEASCo) and grape seed oil (SEASGr)-impregnated stainless steel surface with chemical etching, anodic oxidation and hydrophobizing.

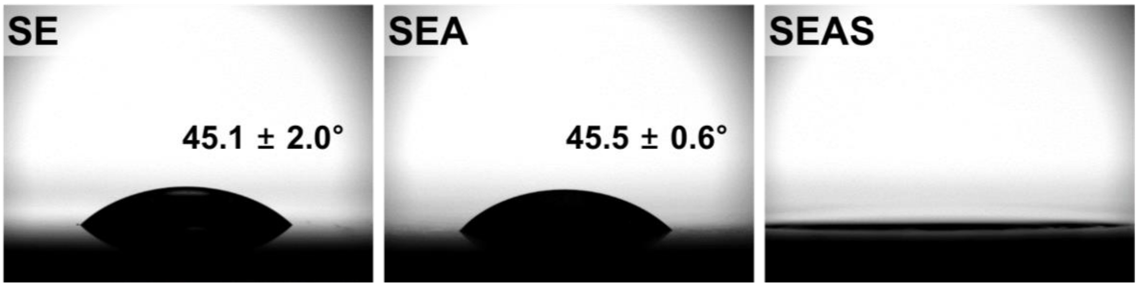

Figure S3. Contact angle of the oleic acid on stainless steel with chemical etching (SE), stainless steel with chemical etching and anodic oxidation (SEA), and stainless steel with chemical etching, anodic oxidation and hydrophobizing (SEAS).

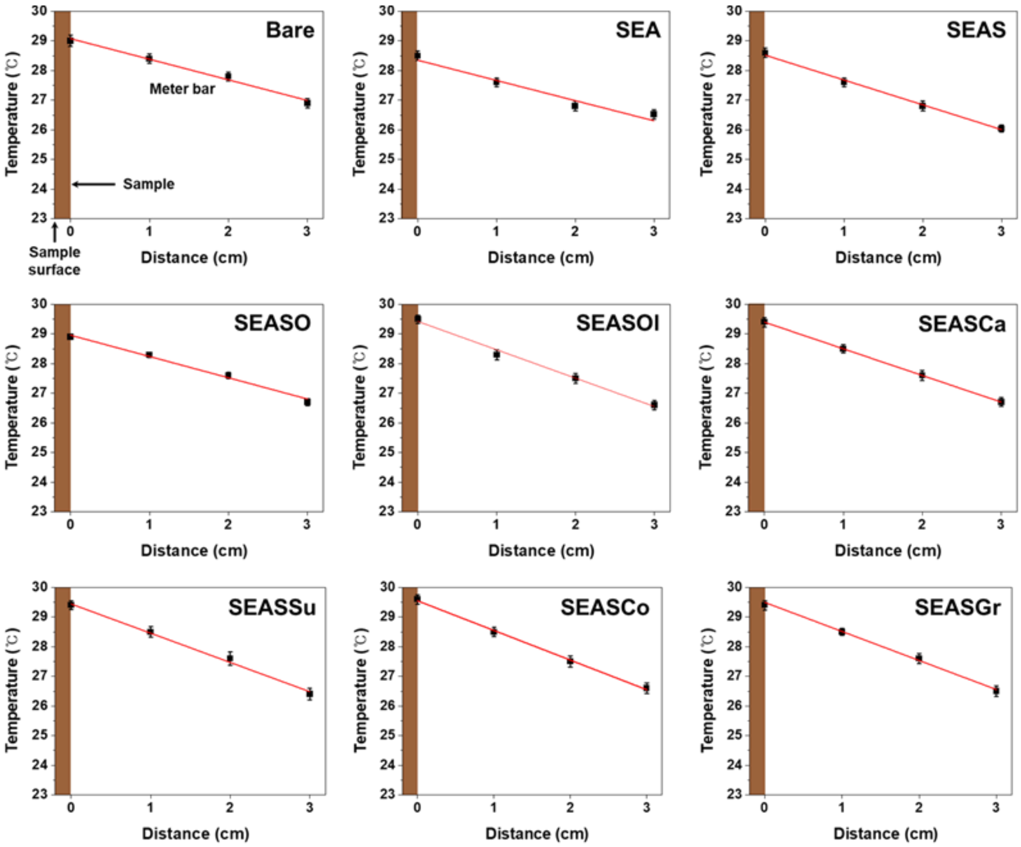

Figure S4. Temperature profile in the meter bar during the condensation test.

Table S1. Estimated temperature gradient from measured temperature in meter bar during condensation heat transfer.

Sample name	Temp. gradient (°C/cm)
Bare	− 0.70
SEA	− 0.68
SEAS	− 0.84
SEASO	− 0.70
SEASOl	− 0.97
SEASCa	− 0.98
SEASSu	− 0.99
SEASCo	− 0.99
SEASGr	− 0.98
